# P01.47. Analgesic and antiedematogenic effect of Energycare™ in the mice model of ischemia-reperfusion of the paw: involvement of anti-inflammatory cytokines

**DOI:** 10.1186/1472-6882-12-S1-P47

**Published:** 2012-06-12

**Authors:** F Cidral-Filho, D Martins, L Mazzardo-Martins, A Soares dos Santos

**Affiliations:** 1 Federal University of Santa Catarina, Florianópolis, Brazil; 2 University of the South of Santa Catarina - UNISUL., Florianópolis, Brazil

## Purpose

Evaluate (1) the possible analgesic and antiedematogenic effect of EnergyCare™ (EC) in the mice model of ischemia-reperfusion of the paw (IR) and (2) the levels of anti-inflammatory cytokines (paw).

## Methods

Male Swiss mice were used in the experiments. All procedures were approved by the University Animal Ethics Comittee (CEUA/Unisul - protocol n# 11.022.4.08.IV). For induction of IR an elastic ring (1.2mm diameter) was positioned in the right ankle of anesthetized mice (chloral hydrate - 7%, 0.6ml/kg, ip). After three hours the ring was cut allowing reperfusion. Experimental groups were: Sham (anesthetized but not subjected to IR), IR, Sham + EC, and IR + EC. For treatment, EC “Dream Pillow” or “Sham Pillow” was placed in the bottom of the animals boxes on the 3rd - removed on the 10th and replaced on the 11th postoperative (PO) day. Mechanical hypersensitivity was determined as response frequency to 10 presentations of a 0.4g von Frey filament. Paw volume was assessed with a plethysmometer. Interleukin (IL)-10 and IL-1ra levels (paw skin and intraplantar muscles) were determined by enzyme-linked immunosorbent assay on the fourth day PO after 24h exposure to EC.

## Results

Two-way ANOVA followed by Bonferroni denoted statistical difference between EC and IR groups in (1) mechanical hypersensitivity [all treatment days (p<0.05 - p<0.001)] and (2) paw volume [PO day 4, 5 (p <0.001) and 6 (p <0.05)]. One-way ANOVA followed by Newman-Keuls indicated increase in the levels of IL-10 [Sham vs Sham + EC (p<0.01), IR vs IR + EC (p<0.05)] as well as IL-1ra (Sham vs Sham + EC and IR vs IR + EC (p<0.05)].

## Conclusion

EnergyCare™ presented analgesic and antiedematogenic effects in the mice model of IR of the paw, as well as induced increase of IL-10 and IL-1ra cytokine levels (paw skin and intraplantar muscles).

